# A C-Terminally Truncated TDP-43 Splice Isoform Exhibits Neuronal Specific Cytoplasmic Aggregation and Contributes to TDP-43 Pathology in ALS

**DOI:** 10.3389/fnins.2022.868556

**Published:** 2022-06-21

**Authors:** Marc Shenouda, Shangxi Xiao, Laura MacNair, Agnes Lau, Janice Robertson

**Affiliations:** ^1^Tanz Centre for Research in Neurodegenerative Diseases, Toronto, ON, Canada; ^2^Department of Laboratory Medicine and Pathobiology, University of Toronto, Toronto, ON, Canada

**Keywords:** *TARDBP*, TDP-43, amyotrophic lateral sclerosis, frontotemporal lobar degeneration, N-terminal fragment, C-splice variant, TDP-43 short, proteinopathy

## Abstract

Neuronal cytoplasmic aggregation and ubiquitination of TDP-43 is the most common disease pathology linking Amyotrophic Lateral Sclerosis (ALS) and frontotemporal lobar degeneration (FTLD). TDP-43 pathology is characterized by the presence of low molecular weight TDP-43 species generated through proteolytic cleavage and/or abnormal RNA processing events. In addition to N-terminally truncated TDP-43 species, it has become evident that C-terminally truncated variants generated through alternative splicing in exon 6 also contribute to the pathophysiology of ALS/FTLD. Three such variants are listed in UCSD genome browser each sharing the same C-terminal unique sequence of 18 amino acids which has been shown to contain a putative nuclear export sequence. Here we have identified an additional C-terminally truncated variant of TDP-43 in human spinal cord tissue. This variant, called TDP43C-spl, is generated through use of non-canonical splice sites in exon 6, skipping 1,020 bp and encoding a 272 aa protein lacking the C-terminus with the first 256 aa identical to full-length TDP-43 and the same 18 amino acid C-terminal unique sequence. Ectopic expression studies in cells revealed that TDP43C-spl was localized to the nucleus in astrocytic and microglial cell lines but formed cytoplasmic ubiquitinated aggregates in neuronal cell lines. An antibody raised to the unique 18 amino acid sequence showed elevated levels of C-terminally truncated variants in ALS spinal cord tissues, and co-labeled TDP-43 pathology in disease affected spinal motor neurons. The retention of this 18 amino acid sequence among several C-terminally truncated TDP-43 variants suggests important functional relevance. Our studies of TDP43C-spl suggest this may be related to the selective vulnerability of neurons to TDP-43 pathology and cell-subtype differences in nuclear export.

## Introduction

TDP-43 is a highly conserved and ubiquitously expressed 43 kDa protein that was first recognized as a TAR DNA binding protein of the LTR region of HIV-1, involved in the transcriptional repression of HIV-1 expression (Ou et al., 1995; Wang et al., 2004). TDP-43 is a mainly nuclear protein controlling many aspects of RNA metabolism such as transcriptional regulation, alternative splicing and mRNA stabilization and transport (Buratti and Baralle, 2001; Buratti et al., 2001; Tollervey et al., 2011; Ratti and Buratti, 2016; Shenouda et al., 2018; Morera et al., 2019). In most cases of Amyotrophic Lateral Sclerosis (ALS) and Frontotemporal Lobar Degeneration (FTLD), TDP-43 is found to mislocalize from the nucleus to the cytoplasm of affected neurons where it forms ubiquitinated aggregates (Arai et al., 2006; Neumann et al., 2006). The basis of this neuronal vulnerability to TDP-43 pathology is unknown. Biochemical analysis of diseased tissue has revealed a pathological signature for TDP-43 comprising of an abnormally phosphorylated species of ~45 kDa that resolves to the correct molecular weight of 43 kDa upon dephosphorylation, a higher molecular weight smear representative of ubiquitinated TDP-43, and a range of lower molecular weight TDP-43 species (Neumann et al., 2006; Igaz et al., 2008). While some of the proposed mechanisms for the generation of the lower molecular weight TDP-43 species in ALS/FTLD include proteolytic cleavage by caspase-3, calpain, or asparaginyl endopeptidases (Zhang et al., 2007; Dormann et al., 2009; Herskowitz et al., 2012; Yamashita et al., 2012), we and others have shown that such moieties can arise from alternative splicing of the *TARDBP* gene (Wang et al., 2002, 2004; Ayala et al., 2011; Polymenidou et al., 2011; Avendaño-Vázquez et al., 2012; D'Alton et al., 2015; Xiao et al., 2015).

*TARDBP* transcript variants arising from alternative splicing at a high splicing density region within exon 6 have been identified in human and mouse, most of which result in frameshifts and generation of C-terminally truncated TDP-43 moieties, collectively referred to as shortened TDP-43 (sTDP-43; Wang et al., 2002, 2004; D'Alton et al., 2015; Weskamp et al., 2020). Shortened TDP-43 isoforms share the same sequence as full-length TDP-43 (TDP43-FL) but lack large regions of exon 6, which encodes the C-terminal glycine rich region where the majority of ALS disease causing mutations are located (Buratti, 2015). In a recent study, two sTDP-43 transcripts were identified in a human iPSC-derived neuron (iNeuron) model that exhibited elevated expression in response to neuronal excitation (Weskamp et al., 2020). The sTDP-43 variants formed cytoplasmic aggregates when endogenously expressed in neuronal culture despite retaining the nuclear localization sequence of full-length TDP-43. To account for this, sTDP-43 variants were found to contain a unique 18 aa sequence at the C-terminus harboring a putative nuclear export sequence (NES) (Weskamp et al., 2020). These findings have shed light on a potential new mechanism of TDP-43 cytoplasmic mislocalization in ALS/FTLD and highlights the importance of characterizing sTDP-43 variants as potential contributors to disease pathogenesis.

Here we have used a nested PCR strategy and 3′ and 5′ Rapid Amplification of cDNA Ends (RACE) to identify an additional sTDP-43 variant in human spinal cord tissue corresponding to EST AU139936 of *TARDBP* (Kimura et al., 2006), and named TDP43C-spl. This variant uses non-canonical splicing sites (AU:AU) and skips region 769–1,788 within exon 6. This splicing event results in a frameshift introducing a downstream stop codon and encodes a 272 aa protein with the first 256 aa identical to human TDP43-FL and a 16 aa sequence at the C-terminus identical to the 18 aa sequence in the sTDP-43 isoforms described previously (Weskamp et al., 2020). To test for cell-type variabilities in subcellular distributions, we demonstrate that both TDP43-FL and TDP43C-spl are localized to the nucleus in astrocytic and microglial cell lines. In contrast, although TDP43-FL maintains a nuclear localization in human and mouse neuronal cell lines, TDP43C-spl is redistributed to the cytoplasm where it forms insoluble ubiquitinated aggregates. Using a novel antibody raised to the C-terminal unique sequence created by the splicing deletion in exon 6, we show upregulation of sTDP-43 variants in ALS tissue and localization to TDP-43 round and skein-like inclusions in disease affected motor neurons. These findings suggest that there is a cell-type specificity related to elevated expression and cytoplasmic localization of shortened TDP-43 variants, which may have implications for neuronal vulnerability in ALS/FTLD.

## Materials and Methods

### Cell Culture

Cell lines were purchased from ATCC and comprised of a mouse microglial cell line, BV2; a human astrocytoma cell line, 1321N1; a human neuroblastoma cell line, SHSY5Y; a mouse neuroblastoma cell line, N2a; and a human kidney cell line, HEK293T. BV2, 1321N1, and HEK293T cells were grown and maintained in DMEM supplemented with 10% FBS. SHSY5Y cells were maintained in DMEM/F12 supplemented with 10% FBS, and N2a cells were maintained in Opti-MEM low serum media (Invitrogen) supplemented with 10% FBS. All cells were transfected with appropriate plasmid DNAs using Lipofectamine 2000 (Invitrogen) following manufacturer instructions.

### RT-PCR and Cloning of Human Full-Length TDP-43 and Its Splice Variant

Total RNA from human dorsal root ganglia (DRG) was obtained from BD Biosciences, Canada. Total RNA from human lumbar spinal cord and human motor cortex was obtained from Ambion Inc. Total RNA from SHSY5Y cells was extracted using TRIzol Reagent (Invitrogen) following the manufacturer's protocol and treated with DNase to minimize DNA contamination. The cDNAs were synthesized from 1 μg of total RNA with Oligo(dT)20 using the SuperScript III First-strand Synthesis System for RT-PCR from Invitrogen, according to the manufacturer's instructions. The mRNA transcripts were first normalized with primers specific for β-actin (Ambion Inc.). For PCR, 2 μl of template cDNA solution was placed in 18 μl of reaction solution containing 10μl of 2 × Master Mix (Applied Biosystems), 6 μl water, 1 μl of 10 μM Forward Primer (5′-GAG AGG ACT TGA TCA TTA AAG GAA TCA G-3′), and 1 μl 10 μM Reverse Primer (5′- TGA ATG AGA AAG CAT GTA GAC AG−3′). Amplification of fragments of TDP-43 and potential splice variants was achieved using a 9800 Fast Thermal Cycler (Applied Biosystems) with an initial denaturation at 95 °C for 10 sec, followed by 35 cycles of denaturation at 95°C for 0 s, and annealing at 66°C for 40 s. The reaction was terminated by a 7 min extension step at 72°C. The PCR products were cloned using the TOPO TA Cloning Kit (Invitrogen) and verified by sequencing. For cloning of human full-length TDP-43 and TDP43-Cspl, 1μg of human lumbar spinal cord total RNA was reverse transcribed with an adapter primer [GGC CAC GCG TCG ACT AGT AC(T)17] to obtain the cDNA, followed by two step nested PCR. For the first round PCR, 1 μl of cDNA was placed in 24 μl of PCR reaction solution containing 1 × PfuUltra buffer, 200 μl dNTPs, 1μl of 10 μM human TDP-43 5′-UTR forward primer (5′-CTG CGC TTG GGT CCG TCG CTG-3′) and 1μl of 10 μM Reverse primer of adapter sequence (5′-GGC CAC GCG TCG ACT AGT AC-3′) and 1.25 U of PfuUltra DNA polymerase (Stratagene). Amplification was achieved with an initial denaturation at 95°C for 45 s, followed by 25 cycles of 95°C for 45 s, 60°C for 45 s and 72°C for 4 min in GeneAmp PCR System 9700 (Applied Biosystems). The PCR product was diluted at 1/100, and subjected to a second round of PCR amplification that utilized a forward primer from the ATG start site including an Xho I restriction enzyme site (5′-CCG CTC GAG CAT GTC TGA ATA TAT TCG GGT AAC-3′) and reverse primer from the 3′-UTR of TDP-43 gene with a Bam HI restriction enzyme site (CGG GAT CCA ACC ACA ACC CCA CTG TCT AC-3′) for full-length of TDP-43, and with forward primer from starting ATG with an Xho I restriction enzyme site (5′-CCG CTC GAG CAT GTC TGA ATA TAT TCG GGT AAC-3′) and reverse primer with a Bam HI restriction enzyme site (5′-CGG GAT CC T TAC AGC ACT ACT TTC AAT G−3′) for TDP43-Cspl. The PCR conditions were identical to the formal amplification process except for a 90 s extension at 72°C. The second PCR products were analyzed by electrophoresis on 1.2 % (w/v) agarose/ethidium bromide gels and purified using the MinElute Gel Extraction Kit (Qiagen), then subcloned into the Xho I/Bam HI sites of pEGFP-C2 or pmRFP (Clontech) and pcDNA3.1(-). Constructs were subjected to sequence analysis for final verification.

### Immunocytochemistry of Cultured Cells

For visualization of EGFP-tagged constructs, cells grown on glass coverslips were fixed with 4% (w/v) paraformaldehyde in phosphate-buffered saline (PBS) at 4°C for 10 min, then washed with PBS and counterstained with DAPI Nucleic Acid Stain (Invitrogen). For ubiquitin labeling, N2a cells were fixed in methanol for 10 min at −20°C, re-hydrated with PBS for 10 min and blocked for 30 min in 5% (w/v) bovine serum albumin with 0.3% (w/v) Triton X-100 in PBS at room temperature. Immunocytochemistry was performed using monoclonal antibody recognizing ubiquitin (mAb1510; Chemicon) at 1:1000 diluted in PBS. Antibody distribution was visualized by epifluorescence microscopy after incubation with secondary antibody conjugated to Alexa 594 diluted at 1:350 in PBS (Molecular Probes), and finally counterstained with DAPI Nucleic Acid Stain. Labeled cells were visualized using a Leica DM6000 microscope and digital images captured using Volocity imaging software (PerkinElmer).

### Protein Extraction and Western Blotting

Cells grown on 35 mm^2^ dishes were harvested at 4°C in 1 ml PBS containing protease inhibitor cocktail (Roche). 500 μl of cell suspension was centrifuged at 500 g for 5 min at 4°C in an Eppendorf Centrifuge. The resultant pellet was re-suspended in 100 μl RIPA buffer containing 1% SDS to give a total protein lysate. The remaining half-volume was centrifuged at 500 g for 5 min and the resultant pellet homogenized in 500 μl of low salt buffer (50 mM Tris, pH 7.5, 150 mM NaCl, 5 mM EDTA) containing protease inhibitor cocktail and sedimented at 16,100 × *g* for 10 min at 4°C in an Eppendorf Centrifuge. Supernatants were saved as low salt soluble fraction, and pellets were subjected to an additional step of homogenization in high salt buffer (50 mM Tris, pH 7.5, 750 mM NaCl, 5 mM EDTA and 1% Triton-X100) containing protease inhibitor cocktail followed by centrifugation at 16,100 × *g* for 10 min at 4°C. Supernatants were saved as high salt soluble fraction and remaining pellets were dissolved with urea buffer [7M urea, 2M thiourea, 4% 3-[(3-cholamidopropyl) dimethylammonio]-1-propanesulfonate (CHAPS), 30 mM Tris-HCl, pH 8.5] and saved as urea fraction. SDS sample buffer (10 mM Tris-HCl, pH6.8, 1 mM ethylenediaminetetraacetic acid, 40 mM dithiothreitol, 1% SDS, and 10% sucrose) was added to 20 μg of each sample followed by heating at 100°C for 5 min, except for the urea fraction, which was not heated to avoid carbamoylation of proteins. For immunoblotting, equivalent volumes of samples were loaded onto 10% SDS-PAGE gels and then electroblotted to polyvinyldiflouride (PVDF) membrane. After blocking in 5% skimmed milk powder in TBS-Tween for 1 h at room temperature, the membrane was incubated with anti-TDP-43 polyclonal antibody (Proteintech Group Inc.), or anti-ubiquitin monoclonal antibody (Chemicon) diluted in blocking solution at 1:1000 and incubated at 4°C overnight. Antibody binding was revealed with the ECL detection system (NEN Life Science Products).

### Generation of C-Terminal Unique Sequence (CTUS) Antibody

To detect TDP43C-spl protein as well as the other sTDP43 variants retaining the same C-terminal unique sequence, rabbit polyclonal antiserum was generated against a synthetic peptide corresponding to VHLISNVYGRSTSLKVVL linked to keyhole limpet hemocyanin. This approach did not produce an antigenic response; therefore, we generated a synthetic peptide incorporating 10 amino acids of the glycine rich region followed by 15 amino acids of the unique sequence, corresponding to the sequence in sTDP43-2 (ERSGRFGGNP**VHLISNVYGRSTSLK**; underline sequence from glycine rich domain, bold unique sequence). The antiserum was subsequently affinity purified using the peptide immunogen.

Since this antibody will recognize sTDP-43 variants with the C-terminal unique sequence, including TDP43C-spl, we refer to it as CTUS-antibody. To eliminate cross-reactivity with TDP43-FL, the CTUS-antibody was immunodepleted using EGFP-TDP-43. HEK293T cells were grown and maintained in DMEM supplemented with 10% Hi-FBS. Cells were transfected with EGFP-TDP-43 using Lipofectamine^®^ LTX (15338; ThermoFisher Scientific) grown on 15 cm^2^ plates (Nunc). 24 h after transfection, cells were harvested with 0.8 ml lysis buffer (0.025 M Tris, 0.15 M NaCl, 0.001 M EDTA, 1% (v/v) NP-40, 5% (v/v) glycerol; pH 7.4) with protease inhibitors (78429, Thermo Fisher Scientific). Lysates were centrifuged at 20,000 × *g* for 10 min at 4°C and 0.8 ml of the supernatant was collected and diluted with 3.2 ml of dilution buffer (0.025 M Tris, 0.15 M NaCl, 0.001 M EDTA, 5% (v/v) glycerol; pH 7.4 with protease inhibitors). The diluted supernatant was then incubated with GFP-Trap^®^ magnetic agarose beads (gmta-20; ChromoTek) for 60 min at 4°C to bind EGFP-TDP43, which was subsequently magnetically separated from the rest of the lysate. The CTUS-antibody was then incubated with the GFP-Trap-EGFP-TDP43 complex overnight at 4°C and magnetically separated to obtain CTUS-antibody depleted of any cross reactivity with TDP43-FL.

### Immunofluorescence Staining of Patient Tissue

ALS and non-neurological disease control autopsy cases were obtained through the ALS Clinic at Sunnybrook Health Sciences Center in Toronto. ALS was diagnosed using the revised El Escorial Criteria (Brooks et al., 2000) and informed consent was obtained with approval from the local ethical review board. 5 μm sections of paraffin-embedded human ALS (*n* = 4) and control (*n* = 4) lumbar spinal cord tissue were deparaffinized at 60°C for 20 min on a heat block and incubated in 2 × 5 min washes of xylene and rehydrated through a series of washes in graded alcohol to 75% (v/v). Autofluorescence was reduced using the Maxblock™ Autofluorescence Reducing Reagent Kit (MaxVision) by adding four drops of Reagent A to the tissue for 2–3 min. The slides were then rinsed in 60% (v/v) ethanol for 1 min, dH_2_0 for 5 min, then 2 × 3 min washes in Tris-buffered saline (TBS; 50 mM Tris-HCL [pH 7.6], and 150 mM of NaCl). Antigen retrieval was carried out by pretreating the sections with TE9 buffer (10 mM Trizma^®^ base, 1 mM EDTA, 0.05% (v/v) Tween 20, pH 9.0) at 110°C in a pressure cooker for 15 min. Tissue sections were then permeabilized and blocked with blocking solution (10% (v/v) donkey serum, 3% (w/v) bovine serum albumin, 0.3% (v/v) Triton™ X-100 in TBS) at ambient temperature for 1 h. Primary antibodies: rabbit polyclonal CTUS-antibody (in-house; 1:100); mouse monoclonal anti-TDP-43 (ab104223; 1:100, Abcam); mouse monoclonal anti-neurofilament-H (SMI-32-R, Covance; 1:500) were diluted in Dako antibody diluent (S08-9; Agilent) and incubated with sections overnight at 4°C. After 3 × 2 min washes in TBS with 0.1% (v/v) Tween (TBST), then 5 min in dH_2_O, sections were incubated with Maxblock™ Autofluorescence Reducing Reagent Kit (MaxVision) Reagent B for 5 min. Slides were then rinsed 3 × 2 min with dH_2_0 and incubated with the appropriate Alexa Fluor^®^ 488 or 594 secondary antibodies (Invitrogen, 1:500) in Dako antibody diluent for 40 min at ambient temperature. After 3 × 5 min washes with TBST, slides were mounted with ProLong^®^ Gold antifade reagent with DAPI (P36931; Life Technologies). For antibody competition assay, 3 μm serial sections were used and incubated with CTUS-antibody alone or CTUS-antibody combined with 5 times excess (w/v) blocking peptide.

## Results

### Cloning of a Human TDP-43 Short Isoform

**Figure 1:**
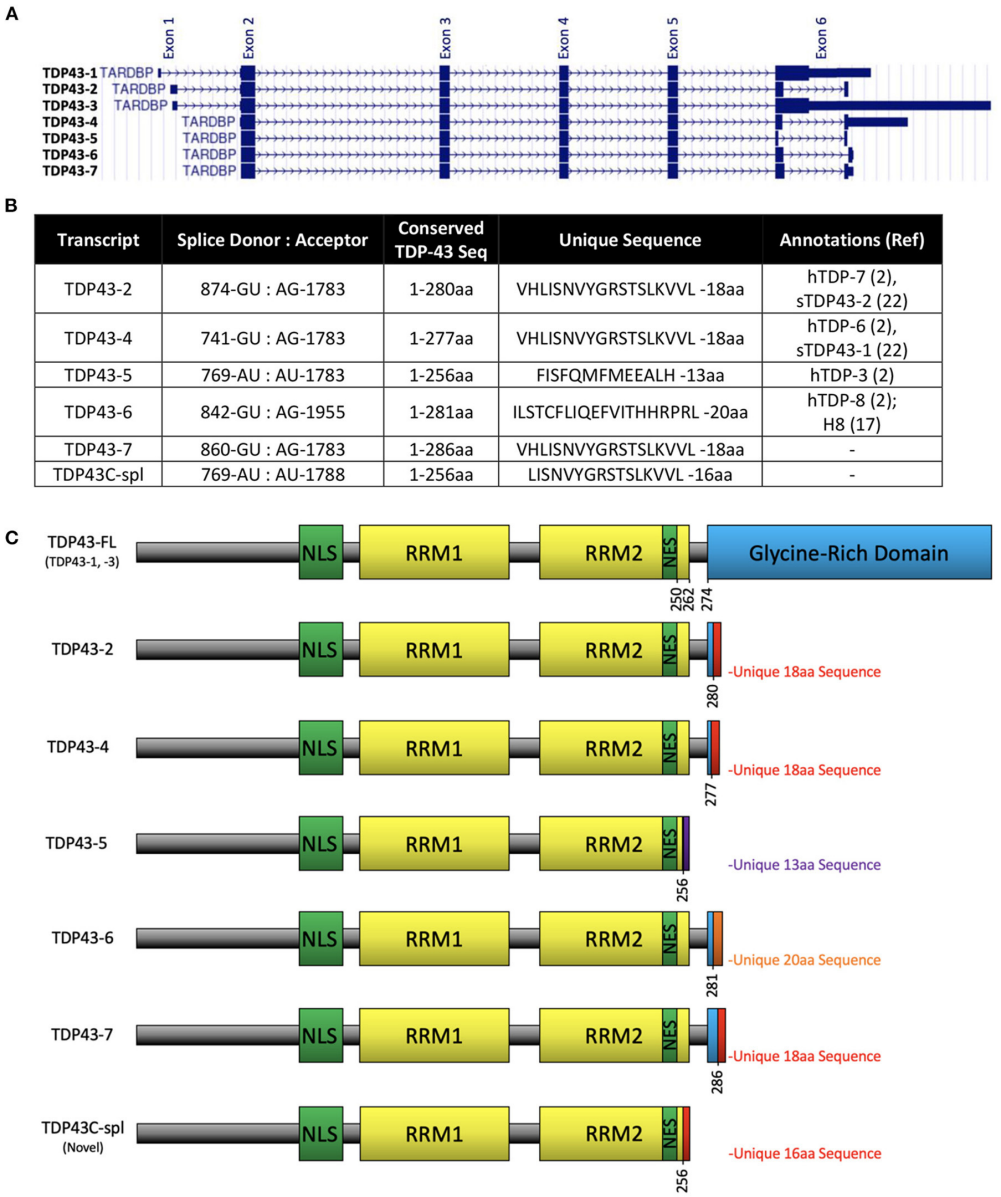
Alternative splice variants transcripts of *TARDBP*. **(A)** Reference assembly of 7 alternate haplotype transcript sequence alignments of *TARDBP* as shown on UCSC genome browser on human (GRCh38/hg38) assembly (https://genome.ucsc.edu) named as TDP43-1 to−7 corresponding to gencode transcripts ENST00000639083.1, ENST00000629725.2, ENST00000240185.8, ENST00000315091.7, ENST00000649624.1, ENST00000616545.4, ENST00000621790.4, respectively. **(B)** Table showing previously identified sTDP-43 and TDP43C-spl splice donor and acceptor dinucleotides, number of conserved amino acids from TDP-43FL, the unique sequences at the C-terminus, and other annotations previously assigned to each isoform. **(C)** Domain structures of TDP-43 protein expressed from each transcript showing the nuclear localization sequence (NLS), RNA recognition motifs (RRM1 and RRM2), nuclear export signal (NES), glycine-rich domain and unique sequences that arise from the alternative splicing events.

**Figure 2:**
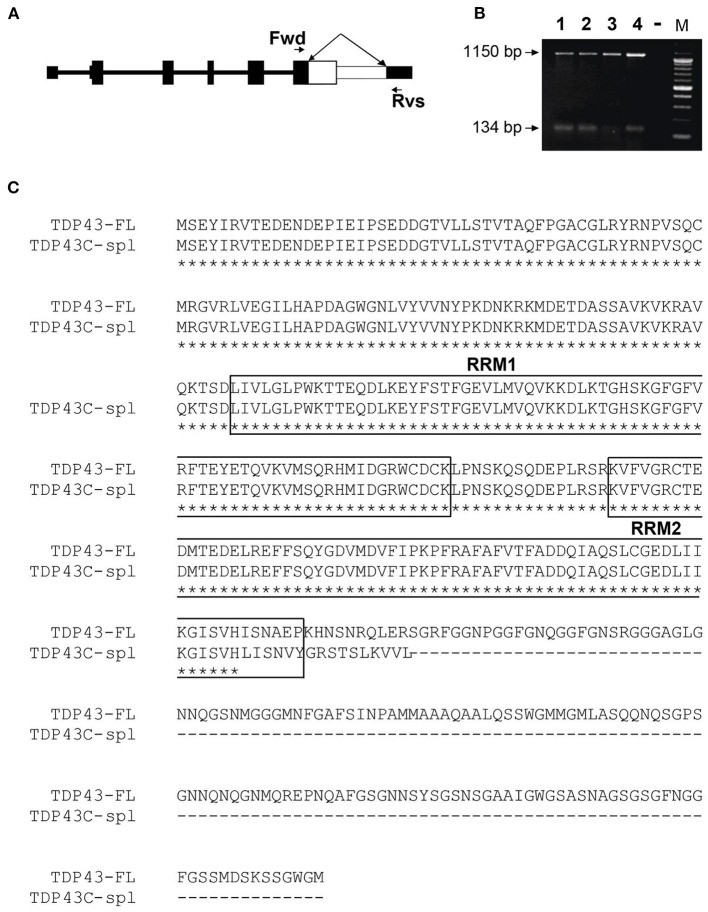
Identification of a human TDP-43 splice variant using RT-PCR. **(A)** Representation of the gene structure of full length TDP-43. The forward (Fwd) primer in CDS of exon 6 and reverse (Rvs) primer in the 3′UTR in exon 6 are indicated by arrows. The white area in exon 6, indicated by the double-headed arrow, represents the region that is spliced out in TDP43C-spl. **(B)** A major higher RT-PCR product of 1,150 bp, corresponding to the human full-length TDP-43 (TDP43-FL), was amplified from RNA isolated from Lane 1, human brain; Lane 2, human spinal cord; Lane 3, human dorsal root ganglion and Lane 4, SHSY5Y cells. The lower band represents a cluster of exon 6 splice variants, with DNA sequencing identifying TDP43C-spl, with a 1,020 bp deletion generating a RT-PCR product of 134 bp. **(C)** Sequence alignment of TDP43-FL and TDP43C-spl. The two RNA-recognition motifs (RRM1 and RRM2) in TDP43-FL are mostly conserved in TDP43C-spl (boxed). A unique 16 aa sequence (LISNVYGRSTSLKVVL) is generated at the C-terminus of TDP43C-spl.

The *TARDBP* gene contains six exons and encodes a 43 kDa protein, TDP-43, comprised of 414 aa (Ou et al., 1995). Human TDP-43 is identical in length to the mouse equivalent, sharing 96% identity and 99% similarity. According to UCSC genome browser (GRCh38/hg38; https://genome.ucsc.edu), human *TARDBP* has seven transcripts, five of which utilize alternative splice sites in the coding region of exon 6 (Figure 1A), resulting in frameshifts and predicting C-terminally truncated protein isoforms. The five isoforms differ in a small number of amino acids retained from full-length TDP-43 due to splice site differences (Figure 1B), three isoforms share the same unique 18 aa sequence at the C-terminus (VHLISNVYGRSTSLKVVL), corresponding to TDP-43 transcripts−2,−4, and−7, using the ordering in UCSC genome browser (Figures 1A–C). It is notable that the sTDP-43 transcripts previously reported equate to TDP-43 transcripts−2 and−4 in Figures 1A–C (Weskamp et al., 2020). We identified a spliced TDP-43 EST AU139936 which indicated an alternative splicing deletion in exon 6. To establish the physiological relevance of this EST, we performed RT-PCR of total RNA from human brain, spinal cord and DRG, and SH-SY5Y cells, using a forward primer located in CDS of exon 6 of human *TARDBP* and a reverse primer in the 3′UTR (Figure 2A). One major product of 1,150 bp was generated by RT-PCR, which corresponded to non-spliced *TARDBP* (Figure 2B). A cluster of species appearing as a single band of ~134 bp was also detected, and sequence analysis confirmed that one of these species was TDP43C-spl generated through use of an alternative splice site in exon 6, skipping 1,020 bp (Figures 2A,B). This splice variant was expressed in brain, spinal cord, DRG and SHSY5Y cells (Figure 2B). Due to the low copy number of the alternative transcript, a nested PCR strategy was employed to obtain the full-length coding region of this alternative transcript called C-terminal splice isoform of TDP-43 (TDP43C-spl). Sequence analysis showed that the transcript of this splice isoform uses non-canonical splicing sites (AU:AU) and skips the region 769–1,788 within exon 6. Translate tool analysis showed that this splice transcript contains a complete ORF with the same ATG start codon as full length TDP-43. It encodes a 272 aa protein with the first 256 aa of TDP43C-spl identical to human TDP43-FL and a unique 16 amino acid sequence at the C-terminus (http://au.expasy.org/tools/dna.html; (Figure 2C). TDP43C-spl encodes RRM1 and most of RRM2 (missing the last 6 amino acids) of TDP43-FL but lacks the C-terminal domain (Figure 2C). After analysis with compute pI/Mw tool (http://ca.expasy.org/tools/pi_tool.html), TDP43C-spl was shown to have a theoretical pI/Mw: 5.54 / 30.53477 kDa. Remarkably the last 18 aa acids of TDP43C-spl, which includes amino acids VH from TDP-43 and LISNVYGRSTSLKVVL unique sequence, is identical to the C-terminal unique sequence in TDP-43 transcripts −2, −4, and −7 (Figures 1B,C). This conserved sequence between 4 different TDP-43 transcripts suggests that it has important functional relevance.

### Subcellular Localization of TDP43C-Spl Within Non-neuronal Origin Cell Lines Is Distinct From That Within Neuronal Origin Cell Lines

**Figure 3:**
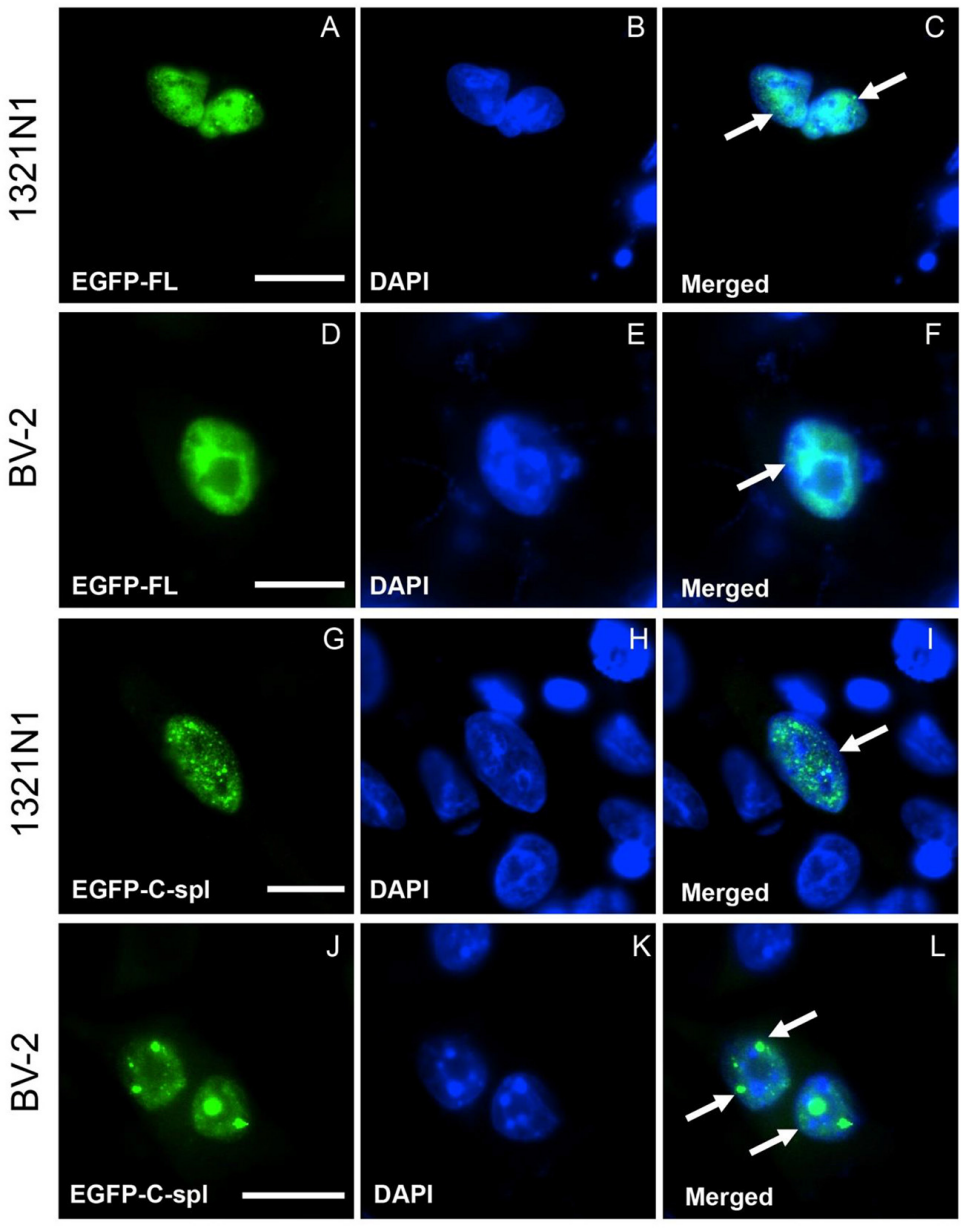
Subcellular localization of TDP43-FL and TDP43C-spl in non-neuronal cells. 1321N1 cells (astrocytoma cell line) and BV-2 cells (microglial cell line) were transfected with EGFP-tagged TDP43-FL **(A–F)** or EGFP-tagged TDP43C-spl **(G–L)**. The EGFP-tagged TDP43-FL **(A,D)** and blue DAPI- nuclear stain **(B,E)** were co-localized, both in 1321N1 cells [arrows in **(C)**] and BV2 cells [arrow in **(F)**]. EGFP-tagged TDP43C-spl **(G,J)** and DAPI-staining **(H,K)** were also co-localized to the nucleus in 1321N1 cells [arrow in **(I)**] and BV2 cells [arrows in **(L)**]. Note that TDP43-FL appears diffusely within nuclei, whereas TDP43C-spl forms speckles [indicated by arrows in **(I,L)**]. Scale bars = 15 μm.

**Figure 4:**
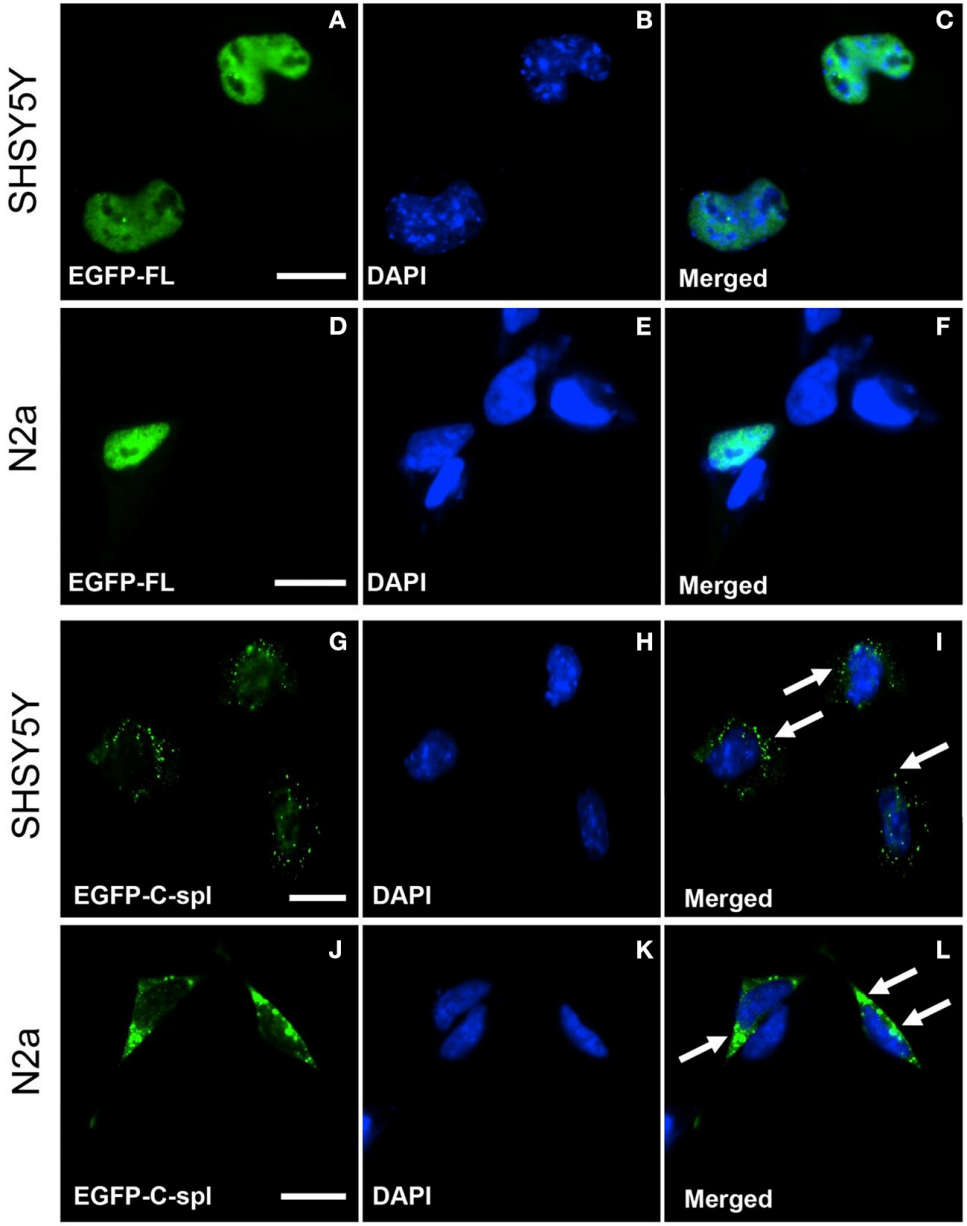
Subcellular localization of TDP43-FL and TDP43C-spl in neuronal cells. SHSY5Y (human neuroblastoma) and N2a (mouse neuroblastoma) cells were transfected with EGFP-tagged TDP43-FL **(A–F)** or EGFP-tagged TDP43C-spl **(G–L)**. The EGFP-tagged TDP43-FL **(A,D)** and blue DAPI nuclear stain **(B,E)** showed co-localization to the nucleus in both SHSY5Y cells **(C)** and N2a cells **(F)**. EGFP-tagged TDP43C-spl was localized to the cytoplasm in both SHSY5Y cells **(G)** and N2a cells **(J)**, with no co-localization with DAPI [**(H,K)**, with overlays shown in **(I,L)**]. Cytoplasmic localization of aggregate TDP43C-spl is shown by arrows in **(I,L)**. Scale bars = 15 μm.

TDP43C-spl retains the nuclear localization and nuclear export sequences of the full-length protein. Expression of EGFP-tagged TDP43C-spl in HEK293T gave nuclear and cytoplasmic localizations (data not shown). To further interrogate the subcellular localization of TDP43C-spl in different cell types, we assessed expression in glial cell lines versus neuronal cell lines (Supplementary Figure 1). For the glial cell lines, we used a human astrocytoma cell line, 1321N1, and a mouse microglial cell line, BV-2. Expression of EGFP-TDP43-FL gave diffuse nuclear localization in both 1321N1 (Figures 3A–C) and BV2 cells (Figures 3D–F). Expression of TDP43C-spl also gave nuclear localization in 1321N1 cells (Figures 3G–I) and in BV2 cells (Figures 3J–L) and formed nuclear speckles which partially co-localized with SC-35, a paraspeckles marker (Supplementary Figure 2). For the neuronal cell lines, we used human SHSY5Y cells and mouse N2a cells. Expression of EGFP-TDP43-FL gave diffuse nuclear localization in SHSY5Y (Figures 4A–C) and N2a cells (Figures 4D–F), similar to that observed for the glial cells. In contrast, EGFP-tagged TDP43C-spl exhibited a striking localization to the cytoplasm in both SHSY5Y and N2a cells, forming aggregates (Figures 4G–L, arrows in I and L). These findings show that the subcellular localization of TDP43C-spl is cell-type dependent, showing nuclear localization in glial cells and forming cytoplasmic aggregates in neuronal cells.

### Cytoplasmic TDP43C-Spl Is Ubiquitinated in Neuronal Cells

**Figure 5:**
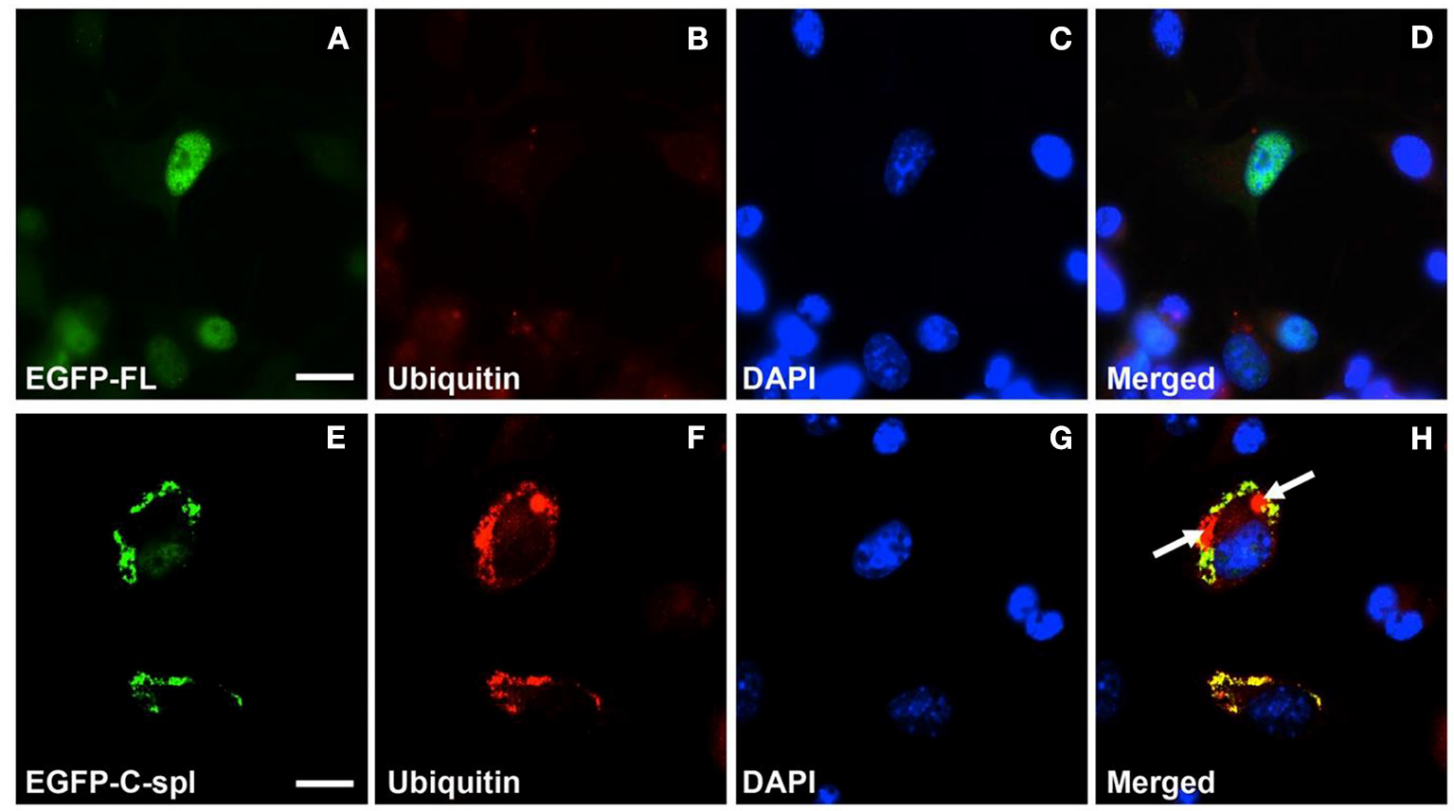
Cytoplasmic aggregates of TDP43C-spl in neuronal cells labeled with ubiquitin antibody. N2a cells were transfected with EGFP-tagged TDP43-FL **(A–D)** or EGFP-tagged TDP43C-spl **(E–H)**. The EGFP-tagged TDP-43 isoforms are shown in **(A,E)**; ubiquitin labeling in **(B,F)**; DAPI stain in **(C,G)**; with the respective merges shown in **(D,H)**. The EGFP-tagged TDP43-FL **(A)** co-localized with blue DAPI-staining nucleus **(C)** was not labeled by anti-ubiquitin antibody **(B,D)**. The EGFP- TDP43C-spl **(E)** and ubiquitin labeling **(F)** were co-localized to the cytoplasm [**(H)**, areas of co-localization appear yellow]. Note regions that are ubiquitin-positive only [indicated by arrows in **(H)**]. Scale bars = 15 μm.

**Figure 6:**
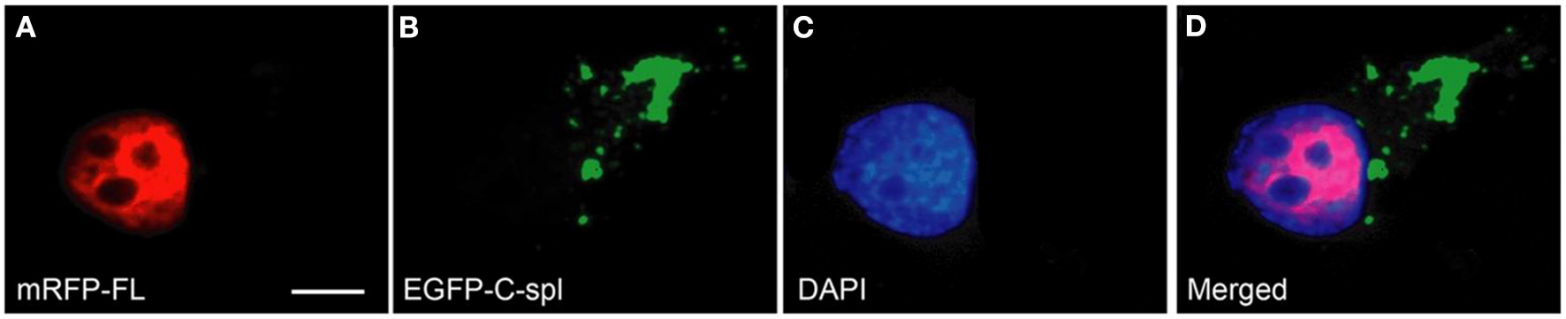
TDP43C-spl aggregates do not recruit TDP43-FL. N2a cells were co-transfected with mRFP-tagged TDP43-FL **(A)** and EGFP-tagged TDP43C-spl **(B)**. Cells were counterstained with DAPI **(C)**. The merged image **(D)** shows that the localization of full length TDP-43 is confined to the nucleus [red in **(A,D)**], whereas TDP43C-spl formed cytoplasmic aggregates [green in **(B,D)**], that were devoid of full-length TDP-43. Scale bars = 30 μm.

To determine if the cytoplasmic aggregates of TDP43C-spl in the neuronal cell lines were ubiquitinated, N2a cells expressing EGFP-tagged TDP43-FL or TDP43C-spl were labeled with ubiquitin antibody. As shown in Figures 5A–D, TDP43-FL localized to the nucleus and was not co-localized with ubiquitin immunoreactivity, which instead gave a low background labeling in some cells (Figure 5B). In contrast the cytoplasmic aggregates of TDP43C-spl were co-localized with ubiquitin labeling (Figures 5E–H), indicating that TDP43C-spl in the cytoplasm but not TDP43-FL in the nucleus becomes ubiquitinated. Interestingly, additional components also appeared embedded in the aggregates formed by TDP43C-spl that were ubiquitin positive but negative for the EGFP signal (shown by arrows in Figure 5H), suggesting that additional factors that potentially interact with TDP43C-spl contribute to the formation of these aggregates. One possibility was that full length TDP43 might be recruited to the cytoplasmic aggregates through associations with TDP43C-spl. To test this, we co-transfected N2a cells with EGFP-tagged TDP43C-spl and RFP-tagged TDP43-FL. As with the single-transfections, TDP43C-spl was localized to the cytoplasm forming aggregates and TDP43-FL was confined to the nucleus (Figure 6). These findings show that there was no recruitment of full-length TDP-43 to the aggregates formed by TDP43C-spl.

**Figure 7:**
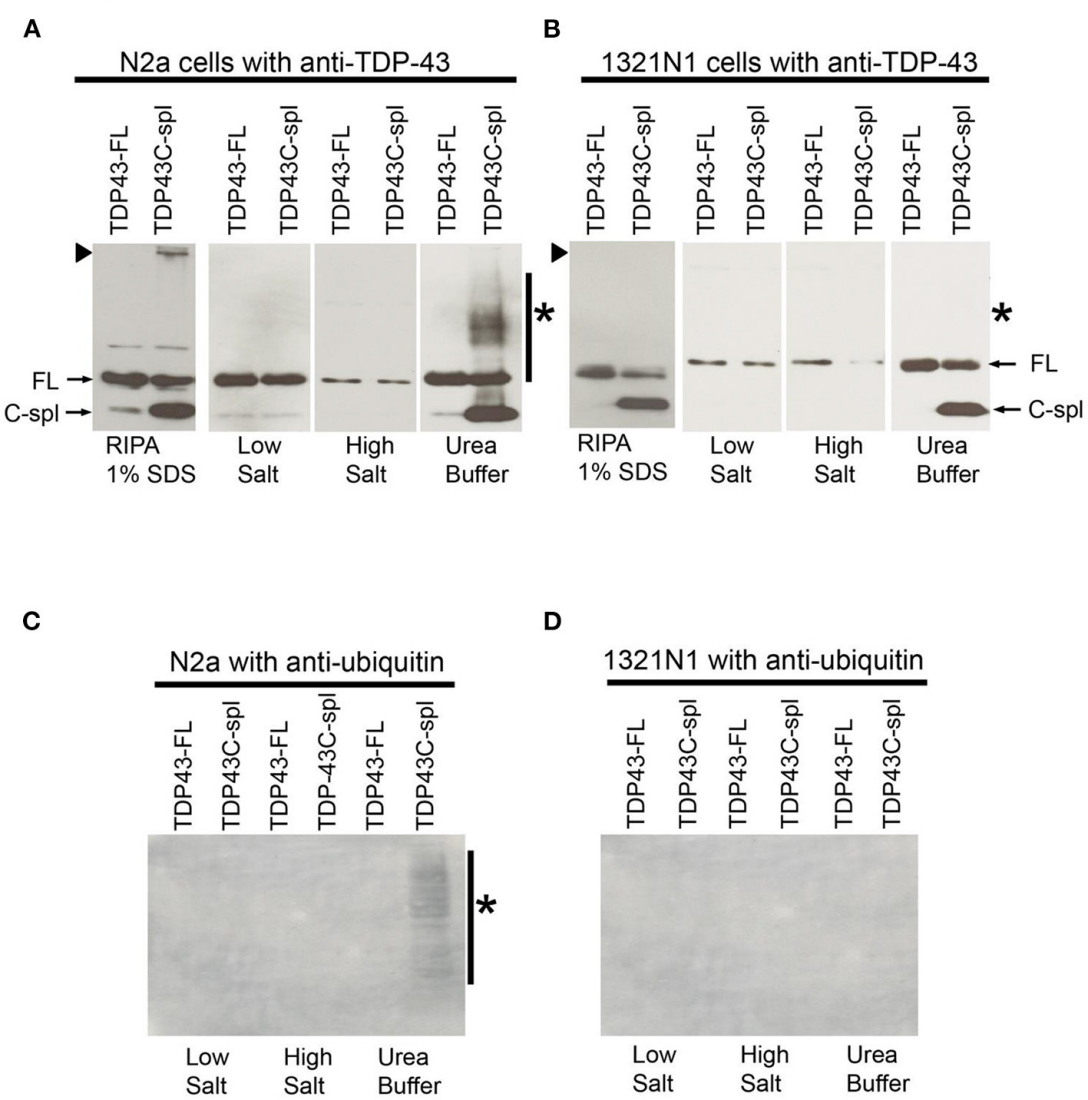
Comparative biochemical analysis of TDP43-FL and TDP43C-spl expressed in N2a and 1321N1 cells. Western blots probed with TDP-43 polyclonal antibody of lysates from N2a cells **(A)** or 1321N1 cells **(B)** expressing TDP43-FL or TDP43C-spl. Bands of 43 kDa correspond to TDP43-FL (FL, indicated by arrow) are detected in all fractions. In TDP43C-spl expressing cells, there was clear expression of the 30 kDa C-spl variant. Bands of ~30 kDa in the N2a cell lysate, but not in the 1321N1 cells, may correspond to endogenous TDP43C-spl (C-spl, indicated by arrow). There is high molecular weight TDP-43 immunoreactive material trapped at the gel interface of N2a cells expressing TDP43C-spl, but not the 1321N1 cells [indicated by arrowhead in **(A,B)**]. The TDP43C-spl partitioned to the urea soluble fraction. High molecular weight material at the gel interface found in the RIPA soluble fraction of N2a cells expressing TDP43C-spl, is no longer apparent and is replaced by a high molecular weight smear (indicated by asterisk in “Urea Buffer”). This smear was absent in the urea soluble fractions from 1321N1 cells expressing TDP43-Cspl. Western blots of samples in **(A,B)** probed with antibody to ubiquitin (C, N2a cells and D, 1321N1 cells). High molecular weight smear shown in the urea soluble fraction of N2a cells expressing TDP43C-spl **(A)** is labeled by ubiquitin antibody in **(C)** (shown by asterisk), with no labeling of any of the samples derived from 1321N1 cells **(D)**.

To biochemically characterize TDP43C-spl protein in neuronal cell lines, immunoblot analysis was performed using cell lysates from N2a cells expressing TDP43-FL and TDP43C-spl. RIPA buffer solubilized pellets from N2a cells expressing TDP43-FL showed a strong 43 kDa band corresponding to full-length TDP-43 (FL in Figure 7A) and a weak ~30 kDa band which might be endogenous TDP43C-spl (C-spl in Figure 7A). Interestingly, RIPA buffer solubilized pellets from N2a cells expressing TDP43C-spl not only showed the 43 kDa and 30 kDa bands, but also higher molecular material trapped at the boundary between the stacking gel and separating gel (Figure 7A, arrowhead). To confirm the identity of the higher molecular material, N2a cells expressing TDP43-FL or TDP43C-spl were sequentially extracted with buffers of increasing salt strength and finally in urea buffer (Figure 7A). Although TDP43-FL was found in all fractions, TDP43C-spl was largely partitioned to the urea soluble fraction, appearing as a band of ~30 kDa, together with a higher molecular weight smear (indicated by asterisk). This smear is likely derived from the RIPA-insoluble material that was trapped at the stacking gel, as this was no longer apparent in the urea-solubilized fractions (Figure 7A).

These experiments were repeated in the 1321N1 cells in which TDP43C-spl is localized to the nucleus (Figure 7B). Interestingly, as with the N2a cells, TDP43C-spl was partitioned to the urea soluble fraction in 1321N1 cells, indicating that urea solubility is not an indicator of the subcellular localization of TDP43C-spl to the cytoplasm (Figure 7B). This was also the case for TDP43-FL, which was present in the urea soluble fraction of both N2A and 1321N1 cells despite being localized to the nucleus. However, no higher molecular weight material was trapped at the interface with the stacking gel in RIPA solubilized 1321N1 cells expressing TDP43C-spl, and no higher molecular weight smear was apparent in the urea soluble fractions (Figure 7B; indicated by arrowhead and asterisk, respectively). This suggests that the higher molecular weight material apparent in lysates of the N2a cells corresponds to cytoplasmic, aggregated TDP43C-spl. Probing of the blots with ubiquitin antibody revealed that the high molecular weight smear present in the urea soluble fraction from N2a cells expressing TDP43C-spl was ubiquitinated (asterisk in Figure 7C), whereas there was no labeling of any of the fractions from the 1321N1 transfected cells (as shown in Figure 7D). These findings support our immunocytochemical data showing that TDP43C-spl is localized to the cytoplasm of N2a cells where it forms ubiquitinated aggregates.

### Upregulation of TDP43C-Spl in ALS and Its Localization to Pathological Inclusions in ALS Motor Neurons by CTUS-Antibody

To determine the pathological relevance of TDP43C-spl and sTDP-43 variants to ALS, we generated an antibody to a synthetic peptide corresponding to the C-terminal 18 aa sequence present in TDP43C-spl and the putative proteins encoded by TDP-43 transcripts−2,−4, and−7 (Figure 1B). We called this antibody CTUS-antibody (C-terminal unique sequence). To eliminate cross-reactivity with TDP43-FL, CTUS- antibody was immunodepleted by incubation with EGFP-TDP-43 bound to anti-EGFP beads. To confirm detection of sTDP isoforms, immunoblots of lysates from N2a cells transiently expressing EGFP-TDP43, EGFP-TDP43-2, or untagged TDP43-2 were probed with the CTUS-antibody or an antibody against EGFP (Supplementary Figures 3A,B). The immunoblots were prepared using cell lysates that were sequentially extracted with buffers of increasing salt strength and finally in urea buffer. The CTUS- antibody recognized EGFP-TDP43-2, or untagged TDP43-2 but not EGFP-TDP43-FL or endogenous TDP-43 (Supplementary Figure 3A), while the EGFP antibody recognized both EGFP-TDP-43FL and EGFP-TDP43-2 (Supplementary Figure 3B). Although EGFP-TDP43-FL was found in all fractions, TDP43-2 was mainly partitioned to the urea soluble fraction, as for TDP43C-spl (Supplementary Figures 3A,B). The same results were found in N2a cells expressing untagged TDP43-2 (Supplementary Figure 3A), indicating that the EGFP tag did not significantly affect protein solubility. These experiments confirmed that CTUS-antibody was specific for the VHLISNVYGRSTSLKVVL sequence and did not cross-react with full-length TDP-43.

**Figure 8:**
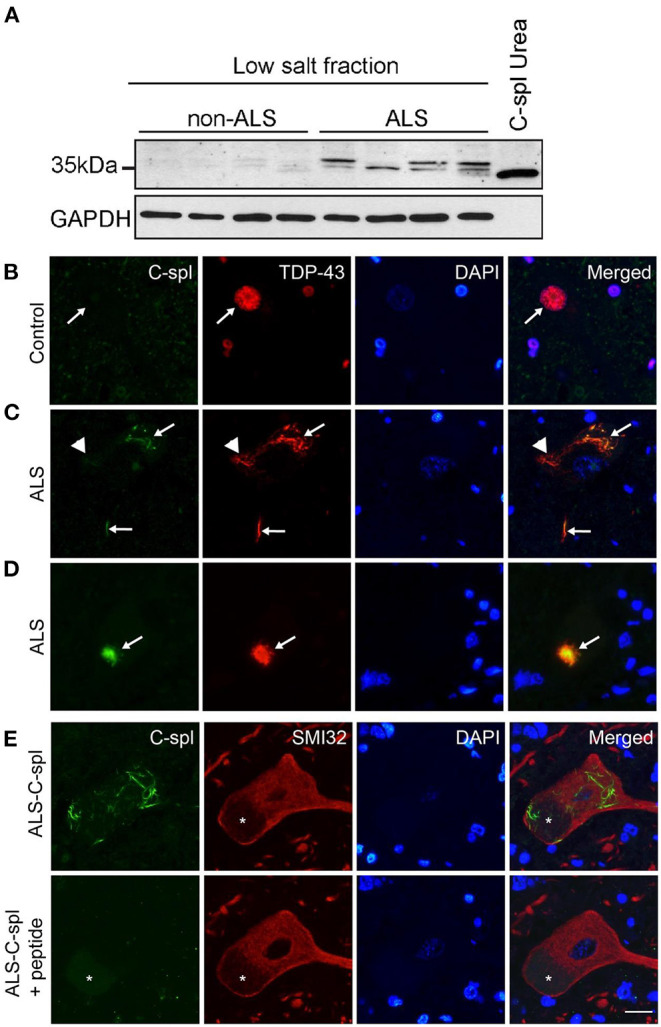
TDP43C-spl/sTDP-43 isoforms are incorporated into TDP-43 positive pathological inclusions in ALS motor neurons. **(A)** Lysates of non-ALS (*n* = 4) and ALS (*n* = 4) lumbar spinal cord show increased expression of TDP43C-spl/sTDP43 in ALS cases compared to controls. GAPDH is used as a loading control and lysate of N2a cells transiently transfected with TDP43C-spl was used as a positive control. Double immunofluorescence staining of spinal cord motor neurons of **(B)** control and **(C–E)** ALS cases with CTUS antibody (green); TDP43-FL (red); and DAPI stain. **(B)** In control cases, there was no apparent CTUS staining in the nucleus (arrows). Note that in **(C,D)** there was co-labeling of CTUS antibody with TDP43-FL in **(C)** skein-like inclusions (arrows), and **(D)** round inclusions (arrows), however this co-labeling was only partial (arrowheads). **(E)** Specificity of labeling was confirmed by competition with the immunizing peptide on serial sections. Motor neurons were identified using non-phosphorylated neurofilament antibody (SMI32, red). Asterisks indicate lipofuscin. Scale bar = 15 μm.

To test for expression of TDP43C-spl and sTDP-43 isoforms in ALS, an immunoblot of lysates from lumbar spinal cord of ALS (*n* = 4) and non-neurological disease controls (*n* = 4) was probed with CTUS-antibody. A range of lower molecular weight species ~30–34 kDa were faintly observed in the control lysates and strongly present in the ALS spinal cord lysates (Figure 8A). This is consistent with the existence of different sTDP-43 isoforms and indicates that they have elevated expression in ALS. Next, we examined whether sTDP-43 isoforms were associated with pathological inclusions in ALS motor neurons. Immunofluorescence labeling using the CTUS-antibody showed that there was no labeling in control spinal cord motor neurons, whereas antibody to TDP-43 labeled the nucleus (Figure 8B, arrow). In ALS cases, CTUS-antibody labeled skein-like (Figure 8C, arrows) and round (Figure 8D, arrow) cytoplasmic TDP-43 positive inclusions, with incomplete overlap (Figure 8D, arrowhead). The specificity of this labeling was confirmed by peptide competition in serial sections with motor neurons identified with neurofilament antibody (SMI32; Figure 8E).

## Discussion

The presence of neuronal cytoplasmic aggregates of TDP-43 is the hallmark pathology of ALS/FTLD. TDP-43 pathology has been linked with N-terminally truncated forms of TDP-43, but C-terminally truncated variants of TDP-43 also exist. Here we have cloned a C-terminal splice variant of TDP-43, called TDP43C-spl, which encodes a 272 aa protein that has the same first 256 aa of TDP-43 and a unique 16 aa C-terminal sequence. This unique sequence is present in three other alternatively spliced transcripts listed in the UCSD genome browser, collectively referred to as sTDP-43. We have demonstrated cell type variabilities in the subcellular distribution of TDP43C-spl in transfected cell lines, with nuclear localization in glial cell lines, and cytoplasmic aggregation in neuronal cell lines. Through generation of a specific antibody, we have demonstrated the upregulation of sTDP-43 protein isoforms sharing the unique C-terminal sequence in lumbar spinal cord tissue from ALS cases versus controls, and that sTDP-43 isoform(s) are integrated in TDP-43 pathology present in spinal motor neurons of ALS cases. This identifies C-terminal splice variants as contributors to TDP-43 pathology in ALS and provides tractable models to investigate cell-type specific mechanisms underlying the cytoplasmic mislocalization of TDP-43.

In the majority of ALS/FTLD cases TDP-43 is mislocalized from the nucleus to the cytoplasm of diseased neurons where it forms ubiquitinated aggregates. N-terminally truncated forms of TDP-43 have been associated with ALS/FTLD, this includes TDP-25 and TDP-35 which may be generated through proteolytic cleavage and/or through abnormal RNA processing events (Dormann et al., 2009; Herskowitz et al., 2012; Yamashita et al., 2012; Xiao et al., 2015). Multiple studies have also identified C-terminally truncated isoforms of TDP-43 (Wang et al., 2002, 2004; Polymenidou et al., 2011; Herskowitz et al., 2012; D'Alton et al., 2015), but their expression at the protein level and relevance to ALS/FTLD pathogenesis have not been fully characterized. In the current study, we identified an alternatively spliced variant of human *TARDBP* corresponding to EST AU139936 in which there is a splicing deletion at a non-canonical splice site (AU:AU) in exon 6, causing a frameshift and introduction of a downstream stop codon. The full-length transcript was cloned from human lumbar spinal cord total RNA and encoded a 272 aa protein with the first 256 aa identical to TDP43-FL and a unique 16 aa sequence at the C-terminus. This novel TDP-43 isoform, nominally called TDP43C-spl, contains RRM1 and most of RRM2 but lacks the C-terminal domain, which harbors most disease causing mutations (Ling et al., 2013; Shenouda et al., 2018; Weskamp et al., 2020). RT-PCR demonstrated that TDP43C-spl was expressed in human spinal cord, brain tissue and DRG. This may be explained by the fact that, like other alternatively spliced genes, the differential expression of the various TDP-43 isoforms may be regulated by the interplay of cis elements and trans factors that exhibit cell type specificities (Chatterjee et al., 2021).

From the UCSC Genome Browser on Human (GRCh38/hg38) Assembly database (https://genome.ucsc.edu), five other C-terminal splice variants have been identified in human *TARDBP*, all of which lack the glycine rich domain. Three of these variants, sTDP43-2, sTDP43-4, and sTDP43-7, encode the same unique C-terminal 18 aa. It is remarkable that TDP43C-spl shares the same C-terminal unique sequence as these variants, indicating the importance of this sequence to the functionality of sTDP-43. In a prior study it was shown that expression of sTDP43-4 in neuronal cells gave cytoplasmic aggregates despite retaining the bipartite NLS of the full-length protein (Weskamp et al., 2020). This was attributed to a potential NES (TSLKV) in the unique 18 aa C-terminal sequence of sTDP43-4. This potential NES is also present in the 16 aa sequence of TDP43C-spl. Although TDP43C-spl formed cytoplasmic aggregates in neuronal cell lines, it retained nuclear localizations in microglial and astrocytoma cell lines. The mechanism by which the presence of this newly identified NES affects protein localization in different cell types remains unknown, but it has been previously shown that some NESs and NLSs can be active in one cell-type and remain non-functional in other cell-types by unelucidated mechanisms (Kosugi et al., 2009; Xu et al., 2012; Lu et al., 2021). In ALS/FTLD, cytoplasmic TDP-43 aggregates occur predominantly within the affected neurons, but they can also occur in oligodendroglial cells (Amador-Ortiz et al., 2007; Higashi et al., 2007; Nakashima-Yasuda et al., 2007; Neumann et al., 2007; Nishihira et al., 2008; Zhang et al., 2008; Yamanaka and Komine, 2018). To our knowledge there are limited reports of cytoplasmic TDP-43 pathology in astrocytes or microglia in ALS/FTLD. Moreover, our current demonstration that TDP43C-spl forms cytoplasmic aggregates in neuronal cells but not in astrocytoma or microglial cells may provide mechanistic insight into cell-type susceptibilities promoting cytoplasmic TDP-43 pathology.

We also demonstrated by immunocytochemistry and immunoblotting that neuronal cytoplasmic aggregates of TDP43C-spl were ubiquitinated. Interestingly, there were foci within the aggregates that were ubiquitin positive but TDP43C-spl negative, indicating that other unidentified factors participate in aggregate formation. This supports our earlier studying showing incomplete overlap of ubiquitin and TDP-43 immunoreactivity in pathological aggregates in motor neurons of ALS spinal cord (Sanelli et al., 2007). Interestingly, there is heterogeneity in the type of ubiquitinated TDP-43 pathology in FTLD, with antibodies recognizing the C-terminus of TDP-43 labeling pathology in Type 1 and Type 2 FTLD, but not Type 3 FTLD (Neumann et al., 2006; Tan et al., 2013; Mackenzie and Neumann, 2017). This suggests variable contributions of N-terminal fragments of TDP-43 across disease types. Although we and others had previously reported recruitment of TDP-43FL to cytoplasmic aggregates formed by truncated forms of TDP-43, we did not observe recruitment of TDP-43FL to cytoplasmic aggregates formed by EGFP-TDP43C-spl. TDP-43 interacts through its N-terminal domain to form homo-oligomers, essential for splicing function (Afroz et al., 2017). As such, it is possible that the EGFP-tag at the N-terminus of TDP43C-spl sterically hindered interaction with the N-terminus of TDP-43, precluding recruitment of full-length TDP-43 to the cytoplasmic aggregates (Weskamp et al., 2020).

To establish the relevance of TDP43C-spl to the pathophysiology of ALS/FTD, we generated a specific antibody recognizing the C-terminal unique sequence (CTUS) shared between TDP43C-spl and the other sTDP-43 variants. This strategy had been reported previously with the antibody generated to the unique sequence able to work for immunohistochemistry but not for immunoblotting. In contrast CTUS-antibody generated herein worked well for both immunohistochemistry and for immunoblotting. Using the CTUS-antibody, we demonstrated the upregulation of sTDP-43 variants in ALS spinal cord lysates compared to controls. We also demonstrated co-labeling of TDP-43 pathology with CTUS-antibody, which is consistent with the prior study, substantiating the contribution of sTDP-43 variants to TDP-43 pathology (Weskamp et al., 2020).

In disease tissues and TDP-43 models of disease, the neuronal splicing profile is highly altered, including abnormal splicing of TDP-43 itself (Arnold et al., 2013; D'Alton et al., 2015; Koyama et al., 2016; De Giorgio et al., 2019; Perrone et al., 2020; Koike et al., 2021). TDP-43 autoregulates its expression presumably by binding to the 3′UTR of its own transcript, which results in splicing of alternative polyadenylation sites that prevents TDP-43 expression through a nuclear retention mechanism (Ayala et al., 2011; Avendaño-Vázquez et al., 2012; Bembich et al., 2014). Numerous studies have shown that TDP-43 autoregulates the level of its own transcript, and some models of TDP-43 transgenic mice have shown a decrease in mTDP-43 levels dependent on the extent of expression of exogenous TDP-43 (Wils et al., 2010; Xu et al., 2010; Igaz et al., 2011; Cannon et al., 2012; De Giorgio et al., 2019). While it has been shown that TDP-43 is able to bind its 3′UTR without its glycine-rich domain (Ayala et al., 2005), that domain is required for autoregulation to occur (Ayala et al., 2011; Avendaño-Vázquez et al., 2012), therefore TDP43C-spl theoretically should not be able to participate in the aforementioned autoregulatory mechanism. Alternatively, it was recently found that DNA demethylation in the autoregulatory region in the *TARDBP* 3′UTR leads to reduced alternative splicing and increased *TARDBP* mRNA expression in human motor cortex (Koike et al., 2021). The exact mechanism leading to the alternative splicing producing TDP43C-spl and the other sTDP-43 variants is yet to be elucidated.

In conclusion, we have characterized a novel C-terminally truncated isoform of TDP-43, TDP43C-spl that is incorporated into pathological inclusions in diseased motor neurons. We have also shown a cell-type dependency for the formation of cytoplasmic ubiquitinated aggregates generated by TDP43C-spl in neuronal cell lines but not in astrocytoma or microglial cell lines. The physiological and pathological functions of TDP-43 splice variants currently remains largely unknown but is an area of great interest. As such, these cell culture models will facilitate investigations into the factor(s) that determine the cell-type specificity of TDP-43 abnormalities, and this may have implications for our understanding of neuronal vulnerabilities in disease.

## Data Availability Statement

The original contributions presented in the study are included in the article/Supplementary Material, further inquiries can be directed to the corresponding author.

## Author Contributions

All authors listed have made a substantial, direct, and intellectual contribution to the work and approved it for publication.

## Funding

This work was supported by funding from the Canadian Institutes of Health Research (#116639) and from the James Hunter ALS Initiative (#471683). MS was funded by a Ph.D. Trainee Award from the ALS Society of Canada in partnership with Brain Canada Foundation and La Fondation Vincent-Bourque (#507996). AL was funded by the Christopher Chiu Fellowship for ALS from ALS Double Play.

## Conflict of Interest

The authors declare that the research was conducted in the absence of any commercial or financial relationships that could be construed as a potential conflict of interest.

## Publisher's Note

All claims expressed in this article are solely those of the authors and do not necessarily represent those of their affiliated organizations, or those of the publisher, the editors and the reviewers. Any product that may be evaluated in this article, or claim that may be made by its manufacturer, is not guaranteed or endorsed by the publisher.
