# Supplementary material for: A C-Terminally Truncated TDP-43 Splice Isoform Exhibits Neuronal Specific Cytoplasmic Aggregation and Contributes to TDP-43 Pathology in ALS
Source: Front Neurosci. 2022 Jun 21;16:868556. doi: 10.3389/fnins.2022.868556 (PMC9253772; doi:10.3389/fnins.2022.868556)
Supplement: Supplementary file 1 [file Data_Sheet_1.docx]

Supplementary Material

**
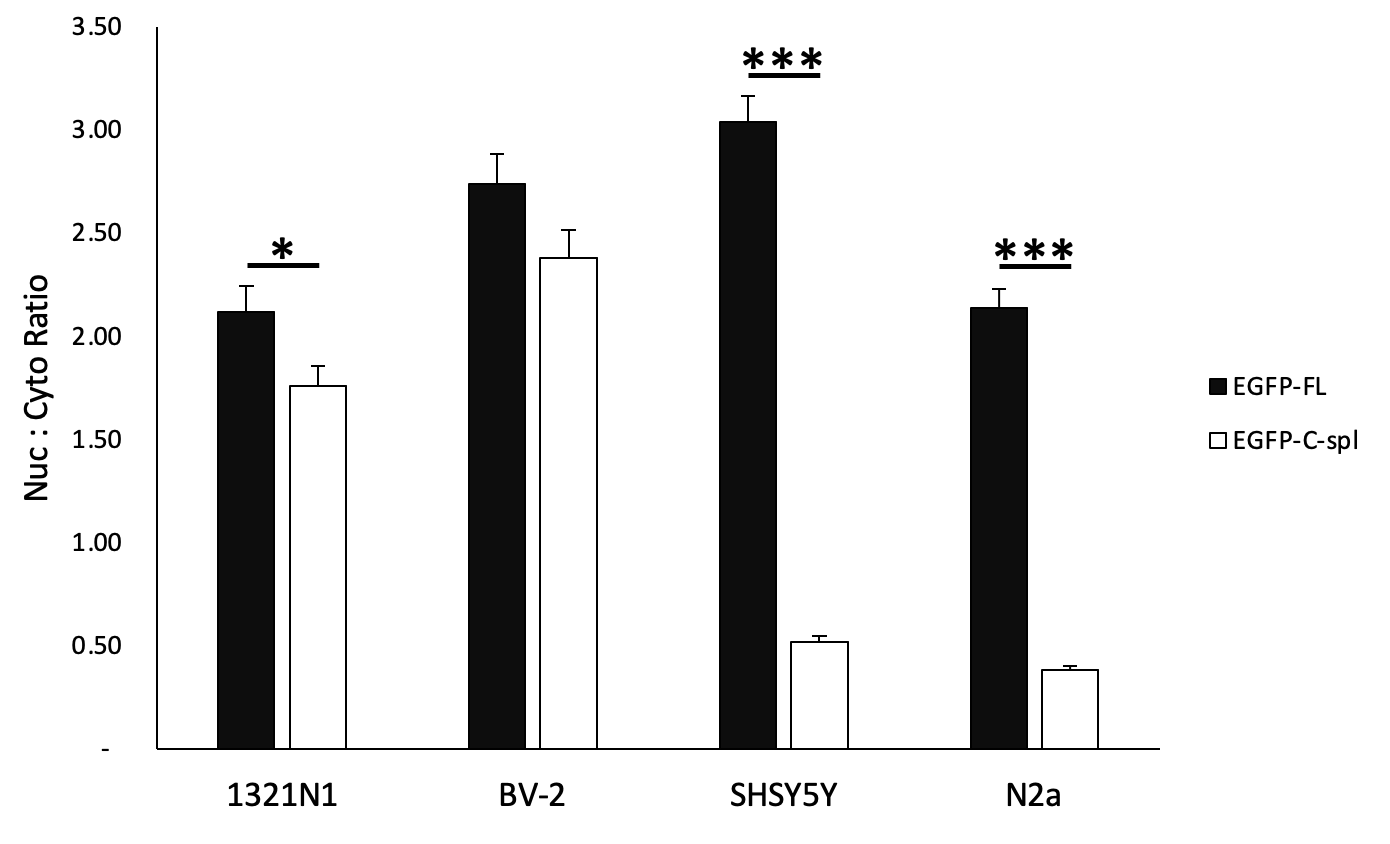
**

**Fig. S1. Nuclear to cytoplasmic ratio of EGFP-TDP43-FL (EGFP-FL) and EGFP-TDP43C-spl (EGFP-C-spl) expressed in microglial, astrocytoma and neuronal cell lines.** EGFP-TDP43-FL retained a mostly nuclear localization in all cell lines (average 2.21 ± 0.12 SEM in 1321N1 cells; 2.74 ± 0.14 in BV-2 cells; 3.04 ± 0.13 in SHY5Y cells; and 2.14 ± 0.09 in N2a cells). EGFP-C-spl had a mainly nuclear localization in astrocytoma cells (average of 1.76 ± 0.10 SEM in 1321N1 cells) and microglial cells (average of 2.38 ± 0.14 SEM in BV-2), and a mainly cytoplasmic localization in neuronal cells (average of 0.52 ± 0.03 SEM in SHY5Y cells; of 0.38 ± 0.02 in N2a cells). Total of 60 cells from 3 biological replicates were analyzed by immunofluorescence for each construct in each cell line. **p<0.5, ** p<0.01, *** p<0.001*


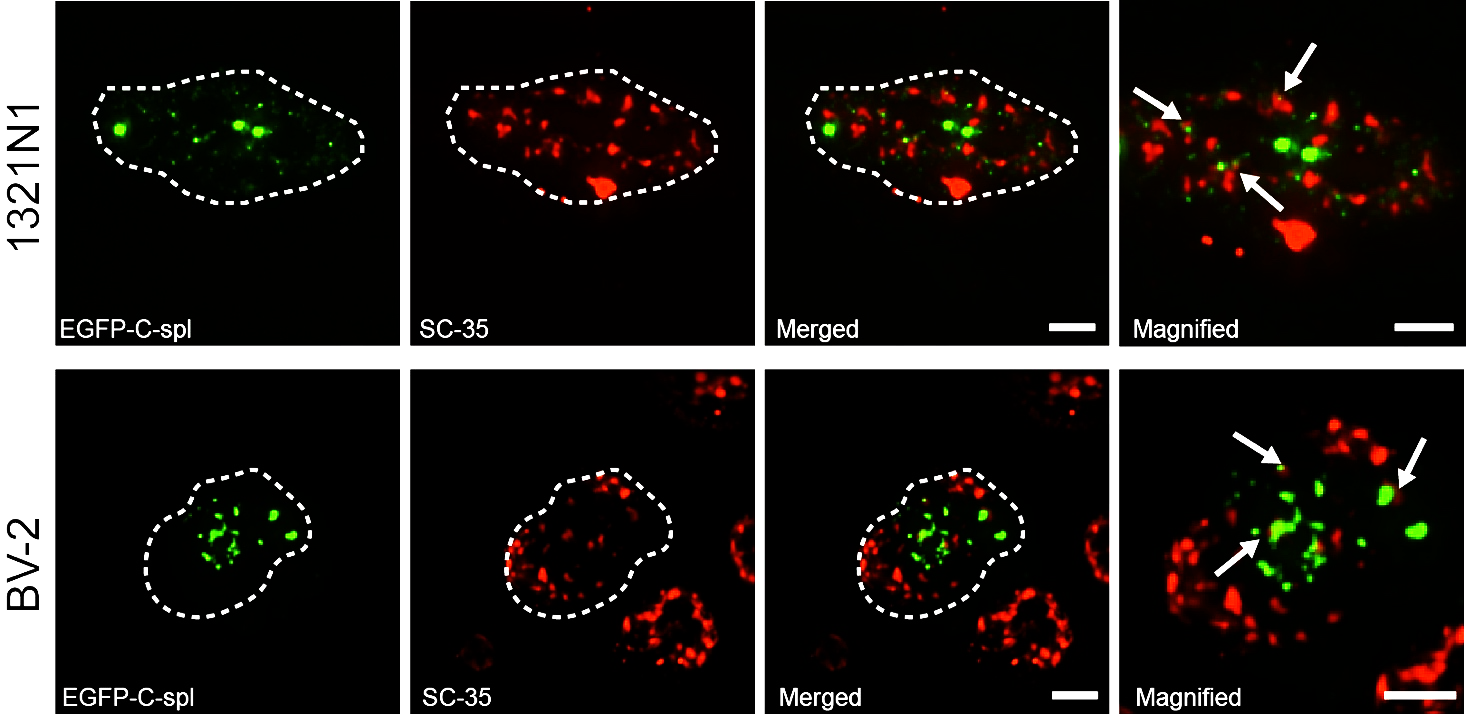


**Fig. S2. Co-localization of TDP43C-spl to paraspeckles in non-neuronal cells.** EGFP-tagged TDP43C-spl show nuclear localization (as outlined based on DAPI staining) in 1321N1 cells (astrocytoma cell line) and BV-2 cells (microglial cell line), and exhibits partial co-localization with SC-35, a paraspeckles marker (indicated by arrows). Scale bars = 2 µm.


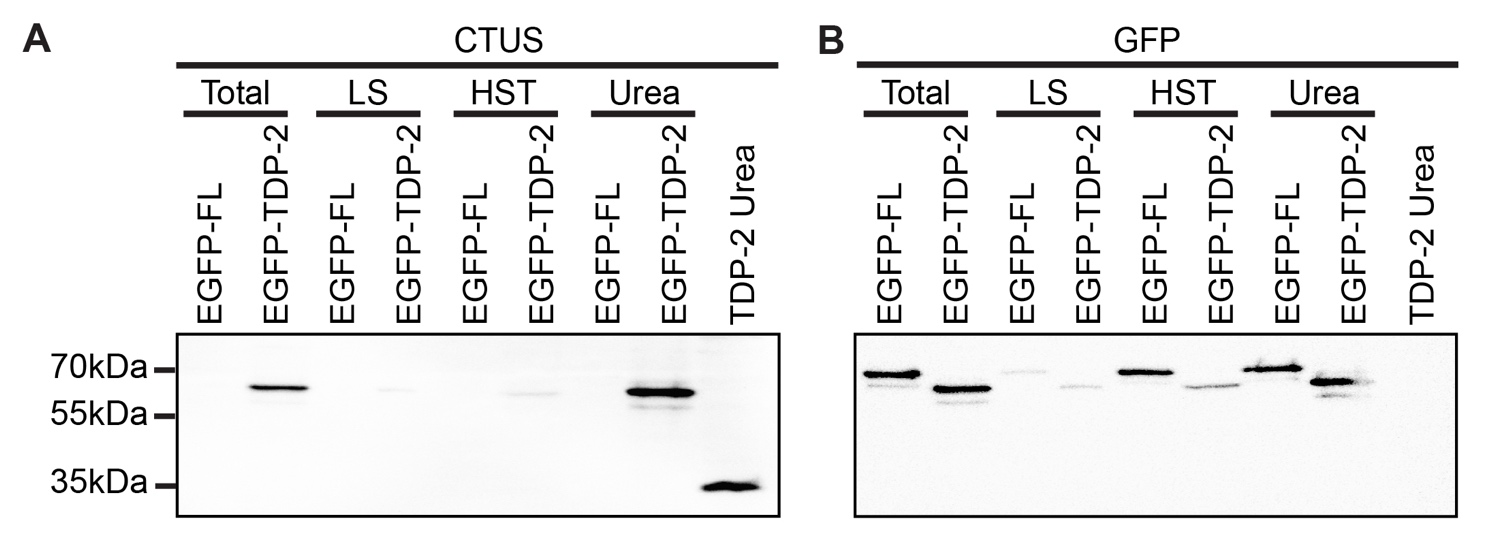


**Fig. S3. Validation of C-terminal unique sequence (CTUS) antibody.** Antibody specificity was confirmed by Western blot analysis of lysates from transfected N2a cells expressing EGFP-TDP43-FL or EGFP-TDP43-2. (A) The CTUS antibody recognized EGFP-TDP43-2 (EGFP-TDP-2) and not EGFP-TDP43-FL (EGFP-FL) in the total protein fraction (RIPA + 2% SDS). (B) EGFP-TDP43-2 was equally as detected by an EGFP antibody as EGFP-TDP-43FL. Protein samples were fractionated in buffers of increasing stringency: low-salt (LS), high-salt Triton™ X-100 (HST) and urea buffer. EGFP-TDP43-2 (EGFP-TDP-2) was mainly found in the urea fraction and slightly in the HST fraction as identified by both CTUS and GFP antibodies, EGFP-TDP-43FL (EGFP-FL) was found in approximately equal amounts in the HST and urea fractions as detected by the EGFP antibody. Urea fraction of N2a cells transiently transfected with untagged TDP43-2 was used as a positive control indicated as TDP-2 Urea.
